# Effectiveness of Natural Frequency Technology^®^ on cognition, sleep, and mood of adults with high perceived stress: A randomized, double‐blind, placebo‐controlled crossover study

**DOI:** 10.1002/brb3.1712

**Published:** 2020-06-08

**Authors:** Heather A. Hausenblas, Stephanie Hooper, Ashlyn Knight, David Hooper

**Affiliations:** ^1^ Center for Health and Human Performance Jacksonville University Jacksonville FL USA

**Keywords:** cognition, mood, Natural Frequency Technology, stress

## Abstract

**Introduction:**

NexQuest Natural Frequency Technology^®^ (NFT^®^), intended to enhance biological function using naturally occurring frequencies, may be a nonpharmacological intervention to improve stress and health. The study purpose was to determine the effectiveness of NFT^®^ for improving stress, sleep quality, mood, and cognition in adults.

**Methods:**

Using a double‐blind placebo‐controlled crossover design, participants with high perceived stress (*N* = 42, *M* age = 43.8) were assessed at baseline (BL) and assigned to either the Placebo Watch (PW) or Wellness Watch (WW) condition for 2 weeks, and then 2 weeks in the alternate condition. Participants completed the following self‐report surveys of Perceived Stress Scale, Pittsburgh Sleep Quality Index, Insomnia Severity Index, Food Craving Questionnaire, and Profile of Mood States, as well as the CNS Vital Signs neurocognitive test at BL and following each condition.

**Results:**

The WW condition had significant improvements in sleep duration and Complex Attention compared to the PW group. Compared to BL, both conditions had significant improvements for perceived stress, food cravings, mood, sleep quality, and several cognitive tests, *p*'s < .05.

**Conclusion:**

Despite the placebo effect, NFT^®^ may be a natural alterative for improving stress and health. Research is needed examining the efficacy of NFT^®^ in a variety of populations and environments.

## Graphical abstract

NexQuest Natural Frequency Technology^®^ (NFT), intended to enhance biological function using naturally occurring frequencies, may be a nonpharmacological intervention to improve stress and overall health. Using a randomized placebo‐controlled design, we found that the NFT resulted in significant improvements in sleep quality, perceived stress, cognition (i.e., Reaction Time, Cognitive Flexibility, Processing Speed, Executive Functioning, and Neurocognition Index), food cravings, and mood for adults who report high perceived stress (*N* = 42). These findings indicate that NFT^®^ may be a safe, natural alterative for reducing stress levels and improving sleep quality, mood, and cognitive performance.

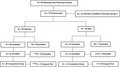

## 1 INTRODUCTION

American adults consistently report elevated stress, with about 1/3 indicating that stress negatively affects their health a lot or to some extent (Keller et al., 2012). More specifically, stress is associated with decreased cognitive ability, mood, and sleep quality and an increased risk of acute and chronic diseases and mortality (Keller et al., 2012; Yaribeygi, Panahi, Sahraei, Johnston, & Sahebkar, 2017). In short, stress is pervasive and causes a variety of diseases as well as decreases in physical, mental, and social health. For example, research has found that perceived stress is related to increased mood issues (e.g., depression and anxiety) and food cravings, and reduced sleep quality and cognitive ability (Barrington, Beresford, McGregor, & White, 2014; Koutsimani, Montgomery, & Georganta, 2019; Moran, 2016; Prather, Bogdan, & Hariri, 2013).

For these reasons, evidence‐based interventions targeting perceived stress are needed. Electromagnetic fields (EMF) influence a range of bodily functions and may represent a novel intervention to reduce stress. EMF sensitivities in cells, plants, animals, and humans have been reported over most frequency ranges, field strengths, and amplitudes occurring in the natural and human‐made EMF environments (Funk, Monsees, & Ozkucur, 2009). In particular, natural EMFs play numerous roles in biological function, and a variety of clinically relevant interactions with the earth's geomagnetic field have been observed (Wiltschko & Wiltschko, 2005).

Clinical research reveals that small fluctuations in weak environmental‐range EMFs have a variety of clinical effects. Sharp or sudden changes in geomagnetic activity (e.g., caused by solar storms) can act as stressors affecting autonomic nervous system activity in people depending on their sensitivity, health status, and capacity for self‐regulation (Alabdulgader et al., 2018). These nanoTesla‐range fluctuations in the ambient magnetic environment are associated with clinically important endpoints such as significant increases in hospital admissions for depression, mental disorders, psychiatric admission, and suicide attempts as well as increases in homicides and traffic accidents (Gordon & Berk, 2003; Halberg, Cornelissen, McCraty, & Al‐Abdulgader, 2011; Halberg, Cornelissen, Panksepp, Otsuka, & Johnson, 2005; Kay, 1994, 2004; Nikolaev Iu, Rudakov, Mansurov, & Mansurova, 1982; Oraevskiĭ et al., 1998). Disturbed geomagnetic activity has also been reported to exacerbate existing diseases such as significant increases in cardiac arrhythmia, cardiovascular disease, mortality due to myocardial infarctions, blood pressure, epileptic seizures, and changes in blood flow (Caswell, Carniello, & Murugan, 2016; Cornélissen et al., 2002; Doronin et al., 1998; Ghione, Mezzasalma, Del Seppia, & Papi, 1998; Giannaropoulou et al., 2014; Halberg et al., 2011; Malin & Srivastava, 1979; Persinger & Psych, 1995; Stoupel, 1993; Stoupel, Wittenberg, Zabludowski, & Boner, 1995). Notably, all of these clinical conditions are stress‐related, suggesting that human responses to small nanoTesla‐range changes within the ambient EMF environment can produce clinically relevant biological effects via pathways regulating stress responses.

In light of these clinically relevant nonthermal biological effects, this study examined Natural Frequency Technology^®^ (NFT^®^), which is a proprietary blend of natural frequencies that is purported to harness and channel frequencies to improve stress and wellness. A recent randomized double‐blind placebo‐controlled crossover study with adults with insomnia symptoms found that NFT^®^ significantly improved their nighttime sleep quality and daytime mood, anxiety, and perceived stress levels similar to those found in meta‐analyses examining complementary and alternative medicines such as melatonin, valerian, yoga, and meditation (Hooper, Coyle, Hooper, Lynch, & Hausenblas, 2019). Further research is needed, however, examining if NFT^®^ provides a simple noninvasive nonpharmacological intervention to reduce daytime stress and improve overall well‐being. Using a randomized double‐blind placebo‐controlled crossover study, the primary purpose was to examine the stress‐reducing effects of NFT^®^ on healthy adults who experience perceived stress. The secondary purpose was to examine the effects of NFT^®^ on food cravings, sleep quality, mood, and cognitive ability. We hypothesized that the NFT^®^ would improve these primary and secondary health outcomes.

## 2 METHODS

### 2.1 Participants

**FIGURE 1:**
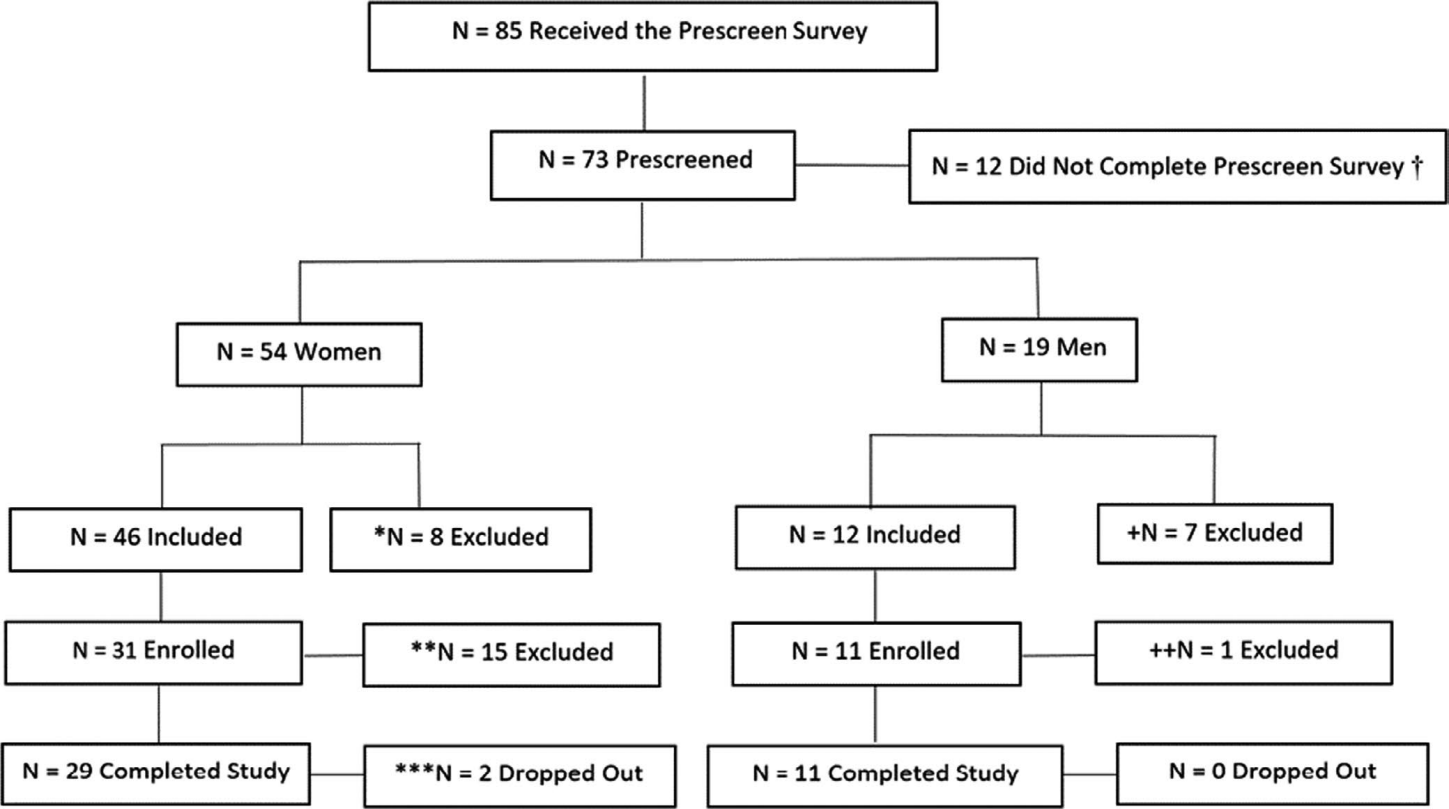
Participant flow chart

Participants were 40 healthy adults (*n* = 11 men and *n* = 29 women, *M* age = 43.8, *SD* = 9.98 years, age range = 26–62 years) who reported experiencing stress. Participants were excluded if they smoked, did not score a 14 or higher on the Perceives Stress Scale (Cohen, Kamarck, & Mermelstein, 1983), had a mental or physical health disorder, or had a BMI greater than 32 (see Figure 1 for the Participant Flow Chart).

### 2.2 Procedures and design

The devices used in this study were Philip Stein wristwatches (Classic Round Large) containing metallic alloy NFT^®^ disks manufactured by NexQuest Life Sciences. The disks were imprinted electromagnetically by exposure to a time‐varying electromagnetic field using a proprietary waveform and method of treatment. This proprietary method imparts a permanent change in the internal composition of the NFT^®^ disks that alters the manner in which the devices interact with weak EMFs such as the geomagnetic field and fields produced by the human body. Maintenance of the delicate electromagnetic equilibrium between living organisms and their natural environment is a key determinant of health and well‐being (Panagopoulos, 2013), and endogenous electric currents and cellular/tissue functions they control can be modulated by externally applied EMFs (Funk et al., 2009).

The Placebo Watch (PW) and Wellness Watch (WW) were indistinguishable from each other and both the participants and the research team were blinded to the conditions (i.e., double‐blinded). Prior to study enrollment the participants signed an Institutional Review Board approved informed consent form. Using a crossover design, and following one week of baseline assessments, the participants were randomized to either the PW or the WW condition for the first two weeks. Then, participants wore the alternative watch for the next two weeks of data collection.

Participants wore the watch from immediately upon awakening until before their nighttime sleep (excluding time spent in water). They received a daily text reminder to wear their watch and to complete questions regarding adherence and adverse events. At PRE (baseline) and POST each condition the participants completed self‐report assessments and performed neurocognitive assessments in a testing room that was free of distractions and extraneous noise. Each testing session took about 45 min. Participants maintained their current lifestyle behaviors for the study duration.

### 2.3 Measures

#### 2.3.1 Food craving questionnaire

The Food Craving Questionnaire is a psychometrically validated measure of the frequency and intensity of general food craving experiences (Meule, Hermann, & Kubler, 2014). This questionnaire assesses the following five dimensions of food craving: (a) intense desire to consume food, (b) anticipation of positive reinforcement that may result from eating, (c) anticipation of relief from negative states and feelings as a result of eating, (d) possible lack of control over eating if food is eaten, and (e) craving as hunger/ physiological state. Participants indicated on a 5‐point scale from 1 (strongly disagree) to 5 (strongly agree) the extent to which they agreed with each item.

#### 2.3.2 Pittsburgh sleep quality index (PSQI)

The PSQI is a reliable and valid instrument used to measure the quality and patterns of sleep in adults. It differentiates “poor” from “good” sleep quality by measuring seven areas: subjective sleep quality, sleep latency, sleep duration, habitual sleep efficiency, sleep disturbances, use of sleeping medications, and daytime dysfunction over the last month (Buysse, Reynolds, Monk, Berman, & Kupfer, 1989).

#### 2.3.3 Insomnia severity index

The Insomnia Severity Index assesses the perceived severity of difficulties initiating sleep, staying asleep, and early morning awakenings, satisfaction with current sleep pattern, interference with daily functioning, noticeability of impairment attributed to the sleep problem, and degree of distress or concern caused by the sleep problem (Bastien, Vallieres, & Morin, 2001).

#### 2.3.4 Profile of mood states (POMS)

The POMS is a psychometrically validated rating scale used to assess the following transient, distinct mood states of tension, anger, vigor, fatigue, depression, and confusion (McNair, Lorr, & Droppleman, 1971). Each item is rated on a 5‐point Likert scale ranging from 0 for feelings they had not experienced, to 5 for extreme feelings.

#### 2.3.5 Perceived stress scale

The Perceived Stress Scale is a measure of the degree to which situations in one's life are appraised as stressful. Items tap how unpredictable, uncontrollable, and overloaded respondents find their lives (Cohen et al., 1983).

#### 2.3.6 CNS vital signs

Cognitive performance was assessed using a validated computerized neurocognitive battery of tests (CNS Vital Signs Inc.) that contains the following seven tests: (a) Verbal Memory that measures recognition memory for WORDS, (b) Visual Memory that measures recognition memory for FIGURES or SHAPES, (c) Finger Tapping which is used to test for motor speed, (d) Symbol Digit Coding which assesses processing speed, (e) Stroop test which measures information processing via congruent and noncongruent associations of color and word, (f) Shifting Attention test that measures ability to shift from differing sets of instructions, and (g) Continuous Performance test that is a measure of sustained attention. Tests were administered in this order (Gualtieri & Johnson, 2006).

The scores obtained by performing the seven tasks generated a composite neurocognitive index and the following 11 domain scores: composite memory, verbal memory, visual memory, psychomotor speed, reaction time, complex attention, cognitive flexibility, processing speed, executive function, and simple attention. The neurocognitive index is a general assessment of the overall neurocognitive status.

### 2.4 Data analysis

The data were examined for normality uses both graphical (histogram) and numerical (skewness, kurtosis, and Shapiro–Wilk) methods. The methods revealed that our data were normally distributed (e.g., Shapiro–Wilk *p*'s > .05). The data were then analyzed using SPSS and Excel to determine condition differences via paired sample *t* tests (*p*'s ≤ .05).

## 3 RESULTS

Two participants withdrew for reasons unrelated to study participation, representing a 95% adherence rate (40/42; see Figure 1). No participants reported an adverse event while wearing either the WW or PW. Overall adherence rate for wearing the watches was 94%.

For the self‐report assessments significant improvements from PRE (baseline) to POST WW and PW were evidenced for PSQI, Insomnia Severity Index, Perceived Stress Scale, Profile of Mood States, and Food Craving Questionnaire, *p*'s < .05 (see Table 1). When participants wore the WW they had significant improvements in sleep duration compared to the PW, *t*(39) = 2.13, *p* = .04.

**TABLE 1:** Descriptive and paired *t* test statistics for the self‐report measures of profile of mood states, perceived stress scale, pittsburgh sleep quality index (PSQI), insomnia severity index, and food craving questionnaire

Outcome	Baseline	PW	WW	Statistic: Paired *t* test		
	Mean (*SD*)	Mean (*SD*)	Mean (*SD*)	Baseline to Watch A	Baseline to Watch B	Watch A to Watch B
Insomnia severity index	12.28 (4.9)	9.65 (5.49)	9.45 (4.67)	*t*(39) = 4.07, *p* < .01	*t*(39) = 4.21, *p *< .01	*t*(39) = 0.28, *p* = .78
PSQI	7.95 (3.46)	6.7 (3.77)	6.53 (3.85)	*t*(39) = 3.11, *p* < .01	*t*(39) = 3.35, *p *< .01	*t*(39) = 0.37, *p* = .71
Profile of mood states	157.3 (13.7)	150.38 (13.84)	151.93 (12.64)	*t*(39) = 3.83, *p *< .01	*t*(39) = 2.80, *p* = .01	*t*(39) = −0.87, *p* = .39
Perceived stress scale	18.7 (5.44)	15.93 (7.31)	16.25 (6.2)	*t*(39) = 3.58, *p *< .01	*t*(39) = 2.65, *p* = .01	*t*(39) = −0.32, *p* = .75
Food craving question	38.48 (15.17)	34.70 (13.87)	35.48 (14.92)	*t*(39) = 2.64, *p* = .01	*t*(39) = 2.20, *p* = .03	*t*(39) = −0.08, *p* = .94

For the CNS Vital Signs, significant improvements from PRE to POST WW and PW were evidenced for Reaction Time, Cognitive Flexibility, Processing Speed, Executive Functioning, and Neurocognition Index, *p*'s < .05 (see Table 2). Significant improvements from PRE to POST WW was found for Complex Attention, *p* = .02. No significant differences were found for Memory, Psychomotor Speed, Verbal Memory, Visual memory, Simple Attention, and Motor Speed, *p*'s > .05.

**TABLE 2:** Descriptive and paired sample *T* test statistics for the CNS vital signs assessments

Outcome	Baseline	Watch A	Watch B	Statistic: Paired *t* test		
	Mean (*SD*)	Mean (*SD*)	Mean (*SD*)	Baseline to Watch A	Baseline to Watch B	Watch A to Watch B
Memory	99.78 (18.23)	100.84 (23.49)	102.29 (22.14)	*t*(36) = −0.27, *p* = .79	*t*(36) = −0.74, *p* = .47	*t*(37) = −0.50, *p* = .62
Psychomotor speed	105.39 (14.88)	108.11 (15.36)	108.37 (15.01)	*t*(37) = −1.47, *p* = .15	*t*(37) = −1.67, *p* = .10	*t*(37) = −0.18, *p* = .86
Reaction time	99.34 (15.63)	105.92 (13.55)	104.58 (12.72)	*t*(37) = −3.11, *p < *.01	*t*(37) = −3.05, *p < *.01	*t*(37) = 0.92, *p* = .36
Complex attention	93.11 (26.58)	96.95 (24.27)	103.11 (10.32)	*t*(37) = −0.76, *p* = .45	*t*(37) = −2.45, *p* = .02	*t*(37) = −1.51, *p* = .14
Cognitive flexibility	99.79 (17.92)	111.89 (13.80)	114.53 (12.31)	*t*(37) = −4.20, *p < *.01	*t*(37) = −6.00, *p < *.01	*t*(37) = −1.21, *p* = .24
Processing speed	105.42 (15.58)	114.21 (15.78)	112.58 (16.39)	*t*(37) = −5.39, *p < *.01	*t*(37) = −3.53, *p < *.01	*t*(37) = 0.99, *p* = .33
Executive functioning	101.05 (17.8)	113.34 (13.41)	115.58 (12.48)	*t*(37) = −4.40, *p < *.01	*t*(37) = −5.75, *p < *.01	*t*(37) = −1.07, *p* = .29
Verbal memory	98.16 (21.12)	98.79 (22.54)	99.79 (20.87)	*t*(37) = −0.18, *p* = .86	*t*(37) = −0.48, *p* = .63	*t*(37) = −0.32, *p* = .75
Visual memory	101.35 (13.83)	102.66 (19.97)	103.79 (18.56)	*t*(36) = −0.50, *p* = .62	*t*(36) = −0.78, *p* = .44	*t*(37) = −0.38, *p* = .71
Simple attention	86.47 (76.8)	81.76 (76.80)	97.63 (12.03)	*t*(37) = 0.41, *p* = .68	*t*(37) = −0.92, *p* = .36	*t*(37) = −1.29, *p* = .21
Motor speed	103.5 (15.83)	101.21 (17.28)	103 (15.37)	*t*(37) = 1.18, *p* = .25	*t*(37) = 0.29, *p* = .77	*t*(37) = −1.00, *p* = .32
Neurocognitive index	99.49 (13.56)	104.71 (13.15)	106.55 (9.72)	*t*(36) = −2.42, *p* = .02	*t*(36) = −2.45, *p <* .01	*t*(37) = −1.06, *p* = .30

## 4 CONCLUSION

This study showed that wearing the WW containing the NFT^®^ resulted in improvements in sleep quality, insomnia symptoms, perceived stress, mood, food cravings, and cognitive outcomes when compared to BL. A placebo effect, however, was noted as the control group also improved on several of the outcomes. Of importance, the WW sleep duration and complex attention improved significantly compared to the PW.

Our study findings provide further support that low frequencies may positively affect health and sleep quality (Breus & Rubik, 2010; Hooper et al., 2019; Jirakittayakorn & Wongsawat, 2018; Thomas, White, Drost, Cook, & Prato, 2001). Natural EMFs play numerous roles in biological function, and a variety of interactions with the earth's geomagnetic field have been observed in a variety of animal species. Clinical effects of either sharp or sudden changes in geomagnetic activity appear to act as stressors affecting autonomic nervous system activity in people in different ways depending on their sensitivity, health status, and capacity for self‐regulation (Alabdulgader et al., 2018). Geomagnetic activity is correlated with daily autonomic nervous system activity in a manner that is synchronized with the time‐varying magnetic field frequencies associated with geomagnetic field‐line resonances and Schumann resonances (Alabdulgader et al., 2018). Geomagnetic disturbances are also associated with clinically important endpoints such as significant increases in hospital admissions for mental health issues (e.g., depression and stress) and cardiovascular issues (Caswell et al., 2016; Gordon & Berk, 2003; Kay, 2004). Further research is needed to examine the effects of NFT^®^ on a variety of health outcomes and its mechanisms of action.

Despite the positive effects found in this study for NFT^®^, a placebo effect was evidenced. Recent research has shown that there is a sizeable placebo effect in research examining the effectiveness of Paroxetine, an antidepressant drug typically prescribed for stress, anxiety, and depression (Kirsch, 2019; Sugarman, Loree, Baltes, Grekin, & Kirsch, 2014). This suggests that psychological conditions like perceived stress respond positively to both active and placebo treatments, possibly explaining why there were similar effects noted in this study.

For the CNS Vital Signs, significant improvements from PRE to POST WW and PW were evidenced for Reaction Time, Cognitive Flexibility, Processing Speed, Executive Functioning, and Neurocognition Index (i.e., general index of overall cognitive status). Significant improvement from PRE to POST WW was found for Complex Attention. Cognitive Flexibility measures how well a person is able to adapt to rapidly changing and increasingly complex set of directions and/or to manipulate the information. Cognitive Flexibility assists with reasoning, switching tasks, decision‐making, impulse control, strategy formation, and attending to conversation. Reaction Time assesses how quickly a person can react to a simple and increasingly complex direction sets. Quick reaction time is relevant, for example, when driving a car, attending to conversation, tracking and responding to a set of simple instructions, and taking longer to decide what response to make. Processing assesses people's ability to recognize and respond/react. Executive Functioning assesses how well a subject recognizes rules, categories, and manages or navigates rapid decision‐making. Finally, Complex Attention measures people's ability to track and respond to a variety of stimuli over lengthy periods and/or perform mental tasks requiring vigilance quickly and accurately.

In conclusion, wearing the WW resulted in improvements in cognitive ability, perceived stress, mood, and sleep quality over a 2‐week period, albeit a significant placebo effect was observed. Thus, further research is needed examining NFT^®^ as an effective, natural, nonpharmalogical way to reduce perceived stress and improve other health outcomes associated with perceived stress compared to placebo. As well, research examining the longitudinal effects of NFT^®^ in a variety of populations and its mechanisms of action underlying the effect are encouraged.

## CONFLICT OF INTEREST

The authors declare no conflict of interest.

## AUTHOR CONTRIBUTION

All the authors contributed to the study design, participant recruitment and retention, and data collection. SH, DH, and HH performed the statistical analyses. HH wrote the manuscript with edits from all the authors.

## Data Availability

The data that support the findings of this study are available from the corresponding author, [HH], upon reasonable request.

## References

[brb31712-bib-0001] Alabdulgader, A. , McCraty, R. , Atkinson, M. , Dobyns, Y. , Vainoras, A. , Ragulskis, M. , & Stolc, V. (2018). Long‐term study of heart rate variability responses to changes in the solar and geomagnetic environment. Scientific Reports, 8(1), 2663 10.1038/s41598-018-20932-x 29422633 PMC5805718

[brb31712-bib-0002] Barrington, W. E. , Beresford, S. A. , McGregor, B. A. , & White, E. (2014). Perceived stress and eating behaviors by sex, obesity status, and stress vulnerability: Findings from the vitamins and lifestyle (VITAL) study. Journal of the Academy of Nutrition and Dietetics, 114(11), 1791–1799. 10.1016/j.jand.2014.03.015 24828150 PMC4229482

[brb31712-bib-0003] Bastien, C. H. , Vallieres, A. , & Morin, C. M. (2001). Validation of the Insomnia Severity Index as an outcome measure for insomnia research. Sleep Medicine, 2(4), 297–307. 10.1016/S1389-9457(00)00065-4 11438246

[brb31712-bib-0004] Breus, M. J. , & Rubik, B. (2010). A randomized, double blind, placebo controlled, crossover evaluation of natural frequency technology™ and sleep natural frequency technology on sleep in normal subjects with un‐refreshing sleep. Sleep Diagnosis and Therapy, 5(5), 27–29.

[brb31712-bib-0005] Buysse, D. J. , Reynolds 3rd, C. F. , Monk, T. H. , Berman, S. R. , & Kupfer, D. J. (1989). The Pittsburgh Sleep Quality Index: A new instrument for psychiatric practice and research. Psychiatry Research, 28(2), 193–213. 10.1016/0165-1781(89)90047-4 2748771

[brb31712-bib-0006] Caswell, J. M. , Carniello, T. N. , & Murugan, N. J. (2016). Annual incidence of mortality related to hypertensive disease in Canada and associations with heliophysical parameters. International Journal of Biometeorology, 60(1), 9–20. 10.1007/s00484-015-1000-3 25913078

[brb31712-bib-0007] Cohen, S. , Kamarck, T. , & Mermelstein, R. (1983). A global measure of perceived stress. Journal of Health and Social Behavior, 24(4), 385–396. 10.2307/2136404 6668417

[brb31712-bib-0008] Cornélissen, G. , Halberg, F. , Breus, T. , Syutkina, E. V. , Baevsky, R. , Weydahl, A. , … Bakken, E. E. (2002). Non‐photic solar associations of heart rate variability and myocardial infarction. Journal of Atmospheric and Solar‐Terrestrial Physics, 64, 707–720. 10.1016/S1364-6826(02)00032-9

[brb31712-bib-0009] Doronin, V. N. , Parfentev, V. A. , Tleulin, S. , Namvar, R. A. , Somsikov, V. M. , Drobzhev, V. I. , & Chemeris, A. V. (1998). Effect of variations of the geomagnetic field and solar activity on human physiological indicators. Biofizika, 43(4), 647–653. 9783072

[brb31712-bib-0010] Funk, R. H. , Monsees, T. , & Ozkucur, N. (2009). Electromagnetic effects ‐ From cell biology to medicine. Progress in Histochemistry and Cytochemistry, 43(4), 177–264. 10.1016/j.proghi.2008.07.001 19167986

[brb31712-bib-0011] Ghione, S. , Mezzasalma, L. , Del Seppia, C. , & Papi, F. (1998). Do geomagnetic disturbances of solar origin affect arterial blood pressure? Journal of Human Hypertension, 12(11), 749–754. 10.1038/sj.jhh.1000708 9844945

[brb31712-bib-0012] Giannaropoulou, E. , Papailiou, M. , Mavromichalaki, H. , Gigolashvili, M. , Tvildiani, L. , Janashia, K. , … Papadima, T. H. (2014). A study on the various types of arrhythmias in relation to the polarity reversal of the solar magnetic field. Natural Hazards, 70, 1575–1587. 10.1007/s11069-013-0890-9

[brb31712-bib-0013] Gordon, C. , & Berk, M. (2003). The effect of geomagnetic storms on suicide. South African Psychiatry Review, 6, 24–27.

[brb31712-bib-0014] Gualtieri, C. T. , & Johnson, L. G. (2006). Reliability and validity of a computerized neurocognitive test battery, CNS Vital Signs. Archives of Clinical Neuropsychology, 21(7), 623–643. 10.1016/j.acn.2006.05.007 17014981

[brb31712-bib-0015] Halberg, F. , Cornelissen, G. , McCraty, R. , & Al‐Abdulgader, A. A. (2011). Time structures (chronomes) of the blood circulation, populations' health, human affairs and space weather. World Heart Journal, 3(1), 1–40.24511305

[brb31712-bib-0016] Halberg, F. , Cornelissen, G. , Panksepp, J. , Otsuka, K. , & Johnson, D. (2005). Chronomics of autism and suicide. Biomedicine & Pharmacotherapy, 59(Suppl 1), S100–108. 10.1016/s0753-3322(05)80017-4 16275478 PMC2576472

[brb31712-bib-0017] Hooper, S. , Coyle, K. , Hooper, D. , Lynch, T. , & Hausenblas, H. (2019). Efficacy of NexQuest Natural Frequency Technology^®^ on health and sleep: A randomized, double blind, placebo controlled crossover trial. Sleep Science. 10.5935/1984-0063.20190091 PMC7347368 32670497

[brb31712-bib-0018] Jirakittayakorn, N. , & Wongsawat, Y. (2018). Brain responses to a 6‐Hz binaural beat: Effects on general theta rhythm and frontal midline theta activity nantawachara. Frontiers in neuroscience, 11, 365 10.3389/fnins.2017.00365 PMC5487409 28701912

[brb31712-bib-0019] Kay, R. W. (1994). Geomagnetic storms: Association with incidence of depression as measured by hospital admission. British Journal of Psychiatry, 164(3), 403–409. 10.1192/bjp.164.3.403 8199794

[brb31712-bib-0020] Kay, R. W. (2004). Schizophrenia and season of birth: Relationship to geomagnetic storms. Schizophrenia Research, 66(1), 7–20. 10.1016/s0920-9964(02)00495-4 14693348

[brb31712-bib-0021] Keller, A. , Litzelman, K. , Wisk, L. E. , Maddox, T. , Cheng, E. R. , Creswell, P. D. , & Witt, W. P. (2012). Does the perception that stress affects health matter? The association with health and mortality. Health Psychology, 31(5), 677–684. 10.1037/a0026743 22201278 PMC3374921

[brb31712-bib-0022] Kirsch, I. (2019). Placebo effect in the treatment of depression and anxiety. Frontiers in Psychiatry, 10, 407 10.3389/fpsyt.2019.00407 31249537 PMC6584108

[brb31712-bib-0023] Koutsimani, P. , Montgomery, A. , & Georganta, K. (2019). The relationship between burnout, depression, and anxiety: a systematic review and meta‐analysis. Frontiers in Psychology, 10, 284 10.3389/fpsyg.2019.00284 30918490 PMC6424886

[brb31712-bib-0024] Malin, S. R. , & Srivastava, B. J. (1979). Correlation between heart attacks and magnetic activity. Nature, 277(5698), 646–648. 10.1038/277646a0 423962

[brb31712-bib-0025] McNair, D. J. , Lorr, M. , & Droppleman, L. F. (1971). Manual for the profile of mood states. San Diego, CA: Educational and Industrial Testing Services.

[brb31712-bib-0026] Meule, A. , Hermann, T. , & Kubler, A. (2014). A short version of the Food Cravings Questionnaire‐Trait: The FCQ‐T‐reduced. Frontiers in Psychology, 5, 190 10.3389/fpsyg.2014.00190 24624116 PMC3940888

[brb31712-bib-0027] Moran, T. P. (2016). Anxiety and working memory capacity: A meta‐analysis and narrative review. Psychological Bulletin, 142(8), 831–864. 10.1037/bul0000051 26963369

[brb31712-bib-0028] Nikolaev Iu, S. , Rudakov, I. , Mansurov, S. M. , & Mansurova, L. G. (1982). Sectoral structure of the interplanetary magnetic field and disturbances of central nervous system activity. Problemy Kosmicheskoi Biologii, 43, 51–59. 7100158

[brb31712-bib-0029] Oraevskiĭ, V. , Breus, T. , Baevskiĭ, R. , Rapoport, S. , Petrov, V. , Barsukova, Z. , … Rogoza, A. (1998). Effect of geomagnetic activity on the functional status of the body. Biofizika, 43(5), 819–826. 9914843

[brb31712-bib-0030] Panagopoulos, D. J. (2013). Electromagnetic interaction between environmental fields and living systems determines health and well‐being In Yoon M. H. K. a. S. O. (Ed.), Electromagnetic fields: Principles, biophysical effects (pp. 107–149). New York, NY: Nova Science Publishers, Inc.

[brb31712-bib-0031] Persinger, M. A. , & Psych, C. (1995). Sudden unexpected death in epileptics following sudden, intense, increases in geomagnetic activity: Prevalence of effect and potential mechanisms. International Journal of Biometeorology, 38(4), 180–187. 10.1007/bf01245386 7601551

[brb31712-bib-0032] Prather, A. A. , Bogdan, R. , & Hariri, A. R. (2013). Impact of sleep quality on amygdala reactivity, negative affect, and perceived stress. Psychosomatic Medicine, 75(4), 350–358. 10.1097/PSY.0b013e31828ef15b 23592753 PMC3747835

[brb31712-bib-0033] Stoupel, E. (1993). Sudden cardiac deaths and ventricular extrasystoles on days with four levels of geomagnetic activity. Journal of Basic and Clinical Physiology and Pharmacology, 4(4), 357–366. 10.1515/jbcpp.1993.4.4.357 8664252

[brb31712-bib-0034] Stoupel, E. , Wittenberg, C. , Zabludowski, J. , & Boner, G. (1995). Ambulatory blood pressure monitoring in patients with hypertension on days of high and low geomagnetic activity. Journal of Human Hypertension, 9(4), 293–294. 7595913

[brb31712-bib-0035] Sugarman, M. A. , Loree, A. M. , Baltes, B. B. , Grekin, E. R. , & Kirsch, I. (2014). The efficacy of paroxetine and placebo in treating anxiety and depression: A meta‐analysis of change on the Hamilton Rating Scales. PLoS One, 9(8), e106337 10.1371/journal.pone.0106337 25162656 PMC4146610

[brb31712-bib-0036] Thomas, A. W. , White, K. P. , Drost, D. J. , Cook, C. M. , & Prato, F. S. A. (2001). A comparison of rheumatoid arthritis and fibromyalgia patients and healthy controls exposed to a pulsed (200 microT) magnetic field: effects on normal standing balance. Neurosci Lett., 309(1), 17–20. 11489536 10.1016/s0304-3940(01)02009-2

[brb31712-bib-0037] Wiltschko, W. , & Wiltschko, R. (2005). Magnetic orientation and magnetoreception in birds and other animals. Journal of Comparative Physiology A Neuroethology, Sensory, Neural, and Behavioral Physiology, 191(8), 675–693. 10.1007/s00359-005-0627-7 15886990

[brb31712-bib-0038] Yaribeygi, H. , Panahi, Y. , Sahraei, H. , Johnston, T. P. , & Sahebkar, A. (2017). The impact of stress on body function: A review. EXCLI Journal, 16, 1057–1072. 10.17179/excli2017-480 28900385 PMC5579396

